# Confirming ERVEBO Vaccination to Support Ebola Virus Surveillance

**DOI:** 10.3201/eid3204.251906

**Published:** 2026-04

**Authors:** Elif Karaaslan, Amy Whitesell, Jason Malenfant, William C. Carson, Michael Townsend, Kasongo Kayembe Jolie, Enogo Koivogui, Siba Michel Grovogui, Boubacar Diallo, Nouonan Gbamou, Salomon Corvil, Sanaba Boumbaly, Lise Martel, Julie R. Sinclair, Alimou Camara, Trevor Shoemaker, Mary J. Choi, Joel M. Montgomery, Christina F. Spiropoulou, Éric Bergeron

**Affiliations:** Centers for Disease Control and Prevention, Atlanta, Georgia, USA (E. Karaaslan, A. Whitesell, J. Malenfant, W.C. Carson, M. Townsend, L. Martel, J.R. Sinclair, T. Shoemaker, M.J. Choi, J.M. Montgomery, C.T. Spiropoulou, É. Bergeron); African Field Epidemiology Network, Conakry, Guinea (K.K. Jolie, S. Corvil); Ministry of Health, Conakry (E. Koivogui, S.M. Grovogui); US Centers for Disease Control and Prevention, Conakry (B. Diallo); Agence Nationale de Sécurité Sanitaire, Conakry (N. Gbamou); International Center for Research of Tropical Infections in Guinea, N’Zerekore, Guinea (S. Boumbaly); Ministry of Higher Education, Scientific Research, and Innovation, Conakry (A. Camara); University of Georgia, Athens, Georgia, USA (É. Bergeron).

**Keywords:** Ebola, Ebola vaccines, Ebola virus disease, Ebola virus infection, vesicular stomatitis Indiana virus, Luminex assay, serologic tests, disease surveillance, vaccine coverage, viruses, United States, Democratic Republic of the Congo

## Abstract

Accurate confirmation of Ebola vaccination (ERVEBO) is essential for interpreting serologic data and assessing vaccine coverage during Ebola virus (EBOV) outbreaks. Current GP1,2-based assays cannot reliably distinguish vaccine-induced immunity from responses generated by natural infection. We developed a multiplex Luminex assay incorporating EBOV GP1,2, secreted glycoprotein (sGP), and a modified vesicular stomatitis virus nucleoprotein (VSV-P-N), a vector antigen encoded by ERVEBO but absent from wild-type EBOV. By using samples from US vaccinees and controls and a small comparison set from the Democratic Republic of the Congo, we found sGP and VSV-P-N demonstrated 100% sensitivity and >97.6% specificity for identifying vaccinees. In samples collected after a ring vaccination campaign in Guinea, combined sGP and VSV-P-N positivity confirmed vaccination in 94.8% of persons with written and 90.8% of persons with verbal confirmation of vaccination history. Our findings show that sGP and VSV-P-N provide a reliable signature of ERVEBO vaccination and support improved Ebola surveillance.

Ebolaviruses, from the Filoviridae family, include 6 known species in the *Orthoebolavirus* genus, 4 of which cause disease in humans: Ebola virus (EBOV), Sudan virus (SUDV), Bundibugyo virus, and Taï Forest virus. Ebola virus disease (EVD) is a rare but severe illness that begins with a nonspecific febrile phase and progresses to gastrointestinal symptoms and multiorgan failure; case-fatality rates are 25%–90% (*1*–*3*). Since 1976, when EBOV was first identified in the northern Democratic Republic of Congo (DRC), ebolaviruses have caused >40 outbreaks, >35,000 cases, and >15,000 deaths (*4*,*5*).

Efforts to control and prevent outbreaks of EVD has led to the development of 2 vaccines, each incorporating the EBOV envelope glycoprotein (GP1,2) as the primary antigen. The recombinant vesicular stomatitis virus (rVSV) vector-based vaccine for EBOV, ERVEBO (Merck, https://www.merck.com), contains the GP1,2 of the EBOV Kikwit strain (*6*). The use of ERVEBO was approved by the US Food and Drug Administration in 2019. The single-dose ERVEBO vaccine demonstrated efficacy by reducing the risk of EVD and death (*7*,*8*). ERVEBO provides protection beginning 10 days postvaccination, with glycoprotein (GP) antibody becoming detectable within 7–10 days (*7*,*9*). The second available vaccine is administered as a heterologous, 2-dose regimen: an adenovirus type 26 vectored vaccine with the GP of EBOV Mayinga variant as the first dose and a modified vaccinia Ankara virus vaccine containing GP of EBOV Mayinga, SUDV Gulu, and Marburg virus Musoke variants and nucleoprotein of Taï Forest virus as the second dose (*10*). Both vaccines induce strong humoral immune responses; the highest titers were detected on day 1 and at the 3-month post booster doses, and by month 12, titers decreased to prebooster levels. Both vaccines induce long-term memory cellular responses and antibody responses that remain detectable for up to 5 years (*11*,*12*).

Several factors lead to an increased risk for EVD outbreaks, including zoonotic spillovers, the expansion of at-risk areas, deficiencies in healthcare systems, and socioeconomic challenges. Among those factors, persistent infections in survivors represents a major concern and has been implicated as the origin of one quarter of outbreaks (*13*–*15*). Although vaccine successes might address many of those challenges, robust and high-sensitivity assays remain essential for accurately determining vaccination status for surveillance and to monitor waning vaccine immunity (*16*). Ebola GP1,2 is the favored antigen, albeit with caveats, for detecting immune responses after vaccination and infection; however, there remains a need to distinguish vaccinees from survivors. Incorporating viral antigens that are not present in the vaccines can better identify survivors, yet identifying vaccinees and monitoring vaccine immunity can benefit from assays targeting vector immunity. Assays that successfully identify vector immunity after rVSV vaccination will be a valuable tool, especially because other rVSV-based vaccines, such as those for SUDV, Nipah virus, and Lassa virus, are currently in various phases of preclinical assessments and clinical trials (*17*–*19*). We describe a multiplex Luminex assay (Diasorin, https://us.diasorin.com) that incorporates Ebola GP1,2, Ebola secreted glycoprotein (sGP), and VSV-P-N antigen detection to confirm Ebola vaccination. 

## Materials and Methods

### Ethics Approval

All research activities were performed in compliance with the regulations and policies to protect human participants, including written consent. For the samples from the Centers for Disease Control and Prevention (CDC), written informed consent was obtained, and the study was approved for use in this research by CDC Institutional Review Board (IRB) (approval no. 7302). The DRC specimens were originally collected under separate study protocol approvals from the CDC IRB and the Kinshasa School of Public Health IRB. Specimens were deidentified (CDC IRB protocol no. 7294) with no reidentification key retained, and this activity was determined to be exempt human subjects research under CDC authority (45 C.F.R. §46.104). In Guinea, the study was approved by the Comité National d’Ethique pour la Recherche en Santé. Negative control human serum from the United States used in this study were obtained from BioIVT (New York, USA), a commercial biorepository. Written informed consent was obtained from all donors before sample collection, and IRB approval was secured by BioIVT for the procurement and use of the specimens in research. Because the samples were fully anonymized, no additional ethics review was required for their use. All samples were stored at −80°C and heat-inactivated at 56°C for 30 minutes before use.

### Recombinant Protein Production

**Figure 1:**
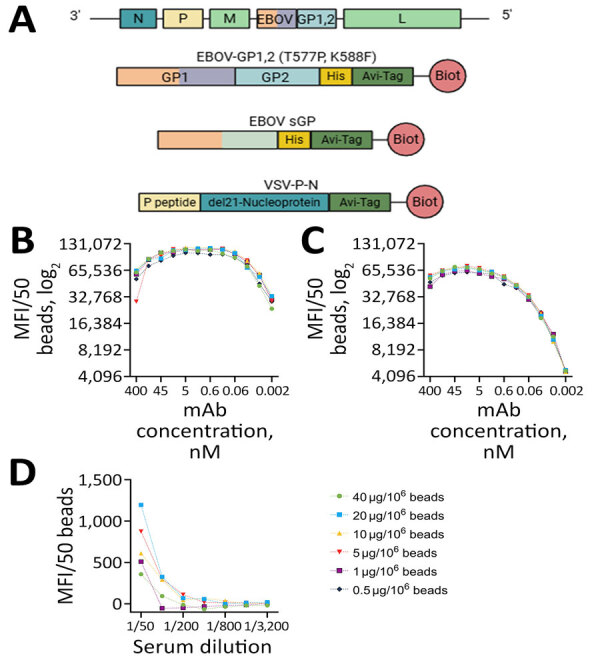
Antigen selection and assay optimization for study of development of multiplex assay to confirm Ebola vaccination. A) Schematic illustration of the rVSV∆G-ZEBOV-GP genome and proteins used in the assay design. Orange indicates the shared portion in GP1 and sGP. del21-nucleoprotein denotes the deletion of the N terminal 21 amino acids of VSV nucleoprotein. B–D) The effect of coupling protein concentration on detection was measured as MFI/50 beads by using mAb114 for EBOV GP1,2 (B) and EBOV sGP (C) and a serum sample from an ERVEBO vaccinee for VSV-P-N (D). Figure created using BioRender (https://www.biorender.com). Avi-tag, Avidin tag; Biot, protein biotinylation; EBOV, Ebola virus; GP, glycoprotein; His-tag, histidine tag; L, large RNA polymerase; M, matrix; mAb, monoclonal antibody; MFI, mean fluorescence intensity; N, nucleocapsid; P, phosphoprotein; P peptide, the first 60 amino acids of the VSV phosphoprotein; rVSV∆G-ZEBOV-GP, recombinant vesicular stomatitis virus where VSV glycoprotein G gene is deleted and replaced with the Ebola virus glycoprotein gene; sGP, secreted glycoprotein; VSV, vesicular stomatitis virus; VSV-P-N, vesicular stomatitis virus nucleoprotein N-terminally fused with P peptide.

We designed the construct for the ectodomain of EBOV GP1,2 (variant Kikwit9; GenBank accession no. P87666) with trimer stabilizing mutations and C-terminal His and Avi-Tags as previously described (Figure 1, panel A) (*20*). We also designed the construct of EBOV soluble GP (variant Ituri2018; GenBank accession no. AYN74068.1) to have C-terminal His and Avi-Tags. We codon-optimized the cDNA sequences for mammalian expression, synthesized, and cloned them into a pEEV-Puro (*21*) episomal expression vector (Twist Bioscience, https://www.twistbioscience.com). To express EBOV GP1,2 and sGP, we grew Expi293F cells (Thermo Fisher Scientific, https://www.thermofisher.com) in Expi293 expression medium (Thermo Fisher Scientific) and transiently transfected them by using the ExpiFectamine 293 Transfection Kit (Thermo Fisher Scientific) following the manufacturer’s recommendations. Four to 6 days after transfection, we collected the culture supernatants, cleared them of cell debris by centrifugation at 10,000 rpm for 20 minutes, and processed them for protein purification.

To increase the solubility, stability, and yield of the nucleoprotein of VSV, we designed a construct with phosphoprotein (P protein) fused to the N-terminus of the nucleoprotein (*22*,*23*). The construct also included an N terminal His-Tag and glutathione S-transferase separated by human rhinovirus 3C cleavage site and a C-terminal Avi-Tag. We codon-optimized the sequence for bacterial expression and cloned into a pET28a bacterial expression vector (Twist Bioscience). The recombinant protein was expressed as described previously (*24*).

We loaded the cleared mammalian cell supernatants and bacterial cell lysates onto HisTrap Excel columns (Cytiva, https://www.cytivalifesciences.com). We biotinylated purified proteins with Avi-Tags by using BirA biotin-protein ligase according to the manufacturer’s instructions (Avidity Biosciences, https://www.aviditybiosciences.com). We removed excess biotin and further purified proteins by using size exclusion chromatography (Superdex 200 pg [Cytiva]). We pooled, aliquoted, and stored protein-containing fractions at −80°C. We produced the cross-neutralizing monoclonal antibody 114 (*25*) in-house by transfecting plasmids encoding the heavy and light chains into Expi293 cells (Thermo Fisher Scientific), and we expressed and purified the monoclonal antibody as described previously (*26*).

### Development of Multiplex Luminex Assay

We used Luminex xMAP (Diasorin) technology to develop the multiplex assay. We bound the biotinylated Avi-tagged EBOV GP1,2, EBOV sGP, and VSV-P-N to avidin-coupled magnetic microspheres (Diasorin), following manufacturer instructions. We removed the storage buffer by magnetic separation and then resuspended the beads in assay buffer (phosphate-buffered saline [PBS; ThermoFisher Scientific] with 1% bovine serum albumin [BSA; Millipore Sigma, https://www.sigmaaldrich.com]). We diluted biotinylated proteins in assay buffer and added them to the microsphere suspension. We incubated the bead-protein mixture for 2 hours at room temperature with rotation. After incubation, we washed the beads 3 times with wash buffer (PBS with 1% BSA and 0.02% Tween-20 [Millipore Sigma]). We counted protein-coupled beads by using the Moxi Z Mini cell counter (ORFLO, https://biofrontiertechnology.com) and stored them in assay buffer at 4°C in the dark. Detailed protocols for assay optimization, validation procedures, and comparison with Filovirus animal nonclinical group (FANG) ELISA standards are provided (Appendix).

### Statistical Analysis

We conducted receiver operating characteristic (ROC) analyses by testing samples from unvaccinated US donors and US vaccinees to determine assay cutoffs, the area under the curve, and the sensitivity and specificity of the assays. We calculated the correlation between assays by using Spearman correlation. We calculated the 95% CIs of the assays percent positivity by using the R package binom (The R Project for Statistical Computing, https://www.r-project.org). We tested the statistical difference in the normalized mean fluorescence intensity (MFI) values in US vaccinees grouped on the basis of time postvaccination by using the Kruskal-Wallis test with the 2-stage step-up method of Benjamini, Krieger, and Yekutieli to correct for multiple comparisons. We determined the statistical significance of the change in the sensitivity of the assays by using Fisher exact test. We performed all analyses by using GraphPad version 10.1.2 (www.graphpad.com) and RStudio (The R Project for Statistical Computing). We created the figures by using BioRender (https://www.biorender.com).

## Results

### Assay Optimization and Internal Reference Standard

Site-specific transcriptional editing of the GP gene of EBOV results in 2 main products: the cleaved structural GP1,2 and the sGP. sGP shares the same N terminal sequence as the GP1 subunit of GP1,2, encompassing the glycan cap and receptor-binding region (*28*). GP1,2 is the trimeric surface protein that enables virus entry and fusion, whereas sGP is produced in large quantities in the plasma of infected patients (Appendix Figure 1). Along with EBOV GP1,2, we investigated EBOV sGP’s performance in serologic assays (Figure 1, panel A). During optimization, we observed a consistent difference between the 2 proteins. Although increasing the coupled protein concentration did not enhance signal intensity for either protein, sGP consistently produced higher signals than GP1,2 at the same monoclonal antibody 114 concentration (Figure 1, panels B, C). Similarly, the use of sGP resulted in higher signals with FANG ELISA reference samples and a serum sample from an ERVEBO vaccinee (Appendix Figure 2, panels A, B). Although the signal with the negative sample (BMI529) was similar (Appendix Figure 2, panels A, B), sGP yielded a 2- to 3.7-fold higher signal with those samples. The higher signal confirmed the observed difference was not limited to monoclonal antibodies but was also possible in polyclonal antibodies. Although that difference might be explained by the larger size of GP1,2, the orientation of the protein during biotin-mediated bead coupling, and epitope accessibility, it also suggests improved separation between negative and positive samples with sGP compared with GP1,2.

As a reference and comparator, we defined the concentration of internal control by using reference and quality control samples in the FANG ELISA to measure Ebola GP1,2 antibodies in vaccine efficacy studies. Our internal reference standard was assigned as 1,024 EU/mL (Appendix Figure 2, panel A).

To express VSV nucleoprotein (VSV-N), we incorporated 2 previously reported modifications into the construct (*22*,*23*). We fused the first 60 amino acids of the P protein to the N-terminus of the VSV-N to prevent N from binding to cellular RNA and self-oligomerization. Next, we expressed VSV-N without the first 21 amino acids, a truncation known to stabilize RNA-bound N oligomers. The design resulted in an RNA-free form of VSV-N, termed VSV-P-N, with the desired solubility and stability (Figure 1, panel A). On the basis of the optimizations, we selected the concentration at which the highest signal was obtained for further assays (Figure 1, panel D). The signal obtained with EBOV-infected nonhuman primate samples was below that of the negative control, whereas FANG ELISA references and ERVEBO vaccinee samples yielded signals 4- to 25-fold higher than the negative control (Appendix Figure 2, panel C).

### Enhanced Detection of ERVEBO Vaccination Status

**Figure 2:**
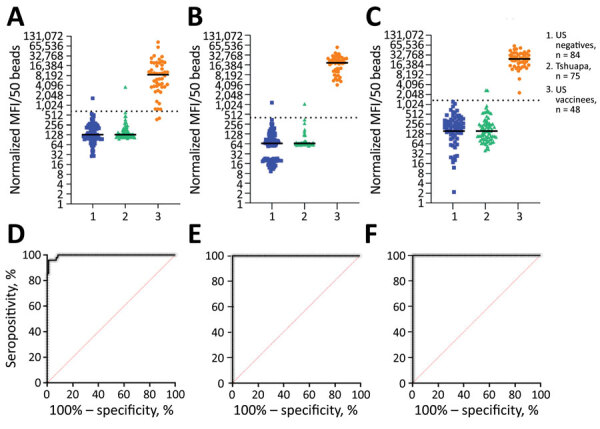
Detection of ERVEBO vaccinees enhanced with EBOV sGP and VSV-P-N for study of development of multiplex assay to confirm Ebola vaccination. A–C) The log_2_-normalized MFI values for EBOV GP1,2 (A), EBOV sGP (B), and VSV-P-N (C) assays for US negative controls (n = 84), Tshuapa samples (n = 75), and US vaccinees (n = 48) are shown in scatter plots. Horizontal solid lines indicate medians; horizontal dotted lines indicate the assay-specific cutoff values. D–F) Receiver operating characteristic (ROC) curves (black lines) and sensitivity and specificity were measured at the defined cutoffs for EBOV GP1,2 (D), EBOV sGP (E), and VSV-P-N (F). ROC area values: D, 0.9950 (95% CI 0.9870–1.000); E, F, 1.000. Sensitivity: D, 95.8% (95% CI 86.02%–99.26%); E, 100% (95% CI 92.59%–100%); F, 100% (95% CI 92.59%–100%). Specificity: D, 98.6% (95% CI 92.83%–99.93%); E, 97.6% (95% CI 91.73%–99.58%); F, 98.8% (95% CI 93.56%–99.94%). EBOV, Ebola virus; GP, glycoprotein; MFI, mean fluorescence intensity; sGP, secreted glycoprotein; VSV-P-N, vesicular stomatitis virus nucleoprotein N-terminally fused with P peptide.

To assess the performance of sGP and VSV-P-N antigens in confirming ERVEBO vaccination status, we determined assay cutoffs by using samples collected from healthy US donors as negatives (n = 84) and from US ERVEBO vaccinees (n = 48). The cutoffs were determined to be 668.7 MFI for EBOV GP1,2 (Figure 2, panels A–D), 400 MFI for EBOV sGP (Figure 2, panels B–E), and 1,350 MFI for VSV-P-N (Figure 2, panels C–F). With those cutoffs, EBOV GP1,2 could detect antibodies in 46 of 48 vaccinees (sensitivity 95.8% [95% CI 86.02%–99.26%], specificity 98.8% [95% CI 92.83%–99.93%]) (Figure 2, panel D; Appendix Figure 3). In comparison, EBOV sGP detected all vaccinees (sensitivity 100% [95% CI 92.59%–100%], specificity 97.6% [95% CI 91.73%–99.58%]) (Figure 2, panel E). Similar to EBOV sGP, VSV-P-N detected all vaccinees, indicating that vector-induced immunity after rVSV vaccination is sufficient as a marker of vaccination status (sensitivity 100% [95% CI 92.59%–100%], specificity 100% [95% CI 93.56%–99.94%]) (Figure 2, panel F). Analysis of correlation between assays revealed a strong correlation between EBOV GP1,2 and EBOV sGP (r = 0.8668; 95% CI 0.7696–0.9248). The correlation between VSV-P-N and EBOV sGP was 0.6080 (95% CI 0.3842–0.7643), whereas the correlation between VSV-P-N and EBOV GP1,2 was 0.5916 (95% CI 0.3621–0.7535) (Appendix Figure 4, panels A–C). Overall, our results reveal the higher sensitivity of EBOV sGP compared with EBOV GP1,2 and underscore the value of including VSV-P-N to identify ERVEBO vaccinees.

Although we used US samples as a negative control group for assay development and cutoff determination, we also evaluated background reactivity by using samples from DRC, an EVD-endemic country that has experienced multiple outbreaks. We included 75 samples collected from healthcare workers in 2017 from the Bokungu and Mondombe territories in Tshuapa Province, DRC. The Tshuapa territories are located outside the Boende health zone, where an EVD outbreak occurred in 2014. This population is considered high risk for natural infection and did not receive an Ebola vaccine before sample collection. Only 1 person from the Tshuapa cohort yielded a signal above the cutoff for both EBOV GP1,2 and sGP, and 2 samples were above the cutoff for VSV-P-N (Figure 2, panels A–C), revealing a 1.3% reactivity for EBOV GP1,2 and sGP and 2.7% reactivity for VSV-P-N in Tshuapa samples.

**Figure 3:**
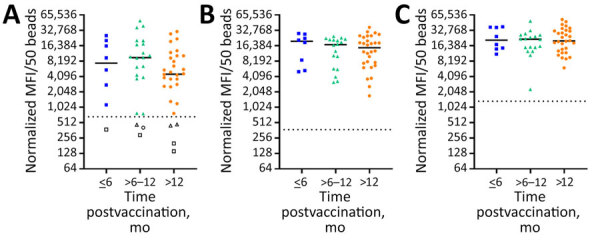
Kinetics of antibody responses in ERVEBO vaccinees for study of development of multiplex assay to confirm Ebola vaccination. US vaccinees who received 1 dose of ERVEBO vaccine were grouped into time intervals of <6 months, >6–12 months, and >12 months postvaccination, and changes in median MFI signal over time were evaluated. A) Ebola virus (EBOV) glycoprotein (GP) 1,2; B) EBOV secreted GP; C) vesicular stomatitis virus nucleoprotein N-terminally fused with P peptide. The scatter plots represent readings from each sample; horizontal solid lines represent group medians, and horizontal dotted lines represent the cutoff for the given protein. The empty squares, triangles, and circle represent samples from 3 vaccinees that were not detected with EBOV GP1,2. MFI, mean fluorescence intensity.

By using those markers to determine vaccination status, we have raised the question of antibody waning and the long-term durability of the immune response. To evaluate this aspect, we grouped samples from the US vaccinees who received 1 dose of the vaccine into time intervals of <6 months, 6–12 months, and >12 months postvaccination and evaluated the changes in median MFI signal over time (Figure 3, panels A–C). Although our sample sizes were limited, we did not detect any major differences in the median MFI signals. However, EBOV GP1,2 did not detect all vaccinees: 3 vaccinees had no positive EBOV GP1,2 at the tested dilution, and 1 additional vaccinee showed discordant results across longitudinal draws (6-month sample detectable by EBOV GP1,2, >12 month sample not detected).

### VSV-P-N and EBOV sGP Identification of ERVEBO Vaccinees After Ring Vaccination Campaign

**Figure 4:**
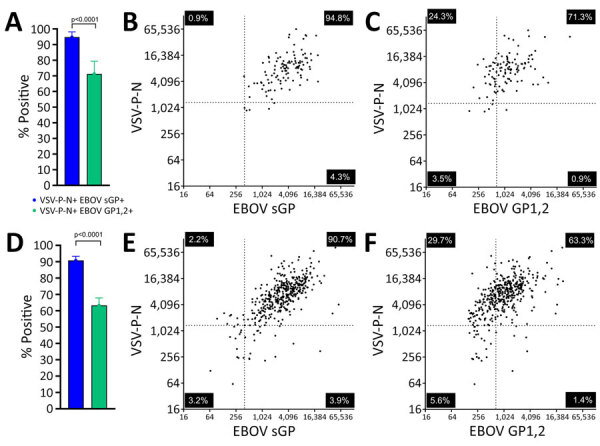
Superiority of EBOV sGP and VSV-P-N in identifying ERVEBO vaccinees for study of development of multiplex assay to confirm Ebola vaccination. A) Percentages of persons with vaccination cards (n = 115) whose vaccination status was confirmed by either positive VSV-P-N and EBOV sGP or positive VSV-P-N and EBOV GP1,2. B, C) Correlation of VSV-P-N and EBOV sGP (B) and VSV-P-N and EBOV GP1,2 (C). D) Percentages of persons with verbally confirmed vaccination status (n = 411) that were confirmed as vaccinees on the basis of positivity for either VSV-P-N and EBOV sGP, or VSV-P-N and EBOV GP1,2. E, F) Correlation of VSV-P-N and EBOV sGP (E) and VSV-P-N and EBOV GP1,2 (F). Error bars indicate 95% CIs. Correlations between results were tested by Spearman correlation r values with 95% CIs. Horizontal and vertical lines in the scatter plots indicate the established cutoff values for the antigens shown on the corresponding axes. Each quadrant of the correlation graphs is labeled with the percentage of the samples that tested negative, single positive, and double positive by assays given on the *x*- and *y*-axes. Figure created using BioRender (https://www.biorender.com). EBOV, Ebola virus; GP, glycoprotein; sGP, secreted glycoprotein; VSV-P-N, vesicular stomatitis virus nucleoprotein N-terminally fused with P peptide.

In 2021, an EVD outbreak was reported with 7 cases in Gouéké, Nzérékoré region, Guinea. A ring vaccination campaign with ERVEBO was initiated for contacts of the EVD cases. Approximately 14 months after vaccination, samples were collected from persons who verbally confirmed EBOV vaccination (n = 411) or presented a vaccination card as written proof (n = 115). Among persons with vaccination cards, 94.8% were positive for both VSV-P-N and EBOV sGP, compared with 71.3% who were positive for both VSV-P-N and EBOV GP1,2 (Figure 4, panel A), a statistically significant difference (p<0.0001). Sensitivity for EBOV GP1,2 was 72.1%, for EBOV sGP was 99.1%, and for VSV-P-N was 96.5% (Appendix Figure 5, panels A–C). The correlation between assays was 0.5687 (95% CI 0.4259–0.6838) for VSV-P-N and EBOV sGP and 0.4266 (95% CI 0.2590–0.5692) for EBOV GP1,2 (Figure 4, panels B, C). Similarly, in the group with only verbal confirmation of vaccination, double-positivity for VSV-P-N and EBOV sGP identified 90.8% of persons as vaccinees, compared with 63.3% with EBOV GP1,2, a statistically significant difference (p<0.0001) (Figure 4, panel D). The correlation between assays was 0.6852 (95% CI 0.6285–0.7346) for VSV-P-N and EBOV sGP, and 0.59 (95% CI 0.5210–0.6513) for EBOV GP1,2 (Figure 4 panels E and F). Consistent with the results from persons who presented a vaccination card, VSV-P-N and EBOV sGP showed strong agreement with a Cohen’s κ coefficient of 0.85 (95% CI 0.79–0.90) compared with VSV-P-N and EBOV GP1,2 (Cohen’s κ coefficient 0.46 [95% CI 0.39–0.53]).

## Discussion

We developed and validated a multiplex Luminex (Diasorin) assay that can simultaneously detect antibodies binding to 3 antigens: EBOV sGP, EBOV GP1,2, and VSV-P-N. Our findings demonstrate robust performance of the assay across different cohorts, including US vaccinees, samples from the Tshuapa province in the DRC, and samples from Guinea with high sensitivity and specificity.

Previous studies in EVD survivors reported 80%–90% seroconversion to GP and sGP, and responses to nucleoprotein (NP) were the next most frequent (*29*,*30*). GP1,2 has been the primary antigen used in serologic assay platforms because it is incorporated into current vaccines and survivors typically exhibit robust seroconversion (*27*,*29*–*34*). However, the use of different cutoff values and reliance on baseline samples to adjust thresholds, weak correlation among GP1,2-based assays, and the need to include multiple antigen targets to improve specificity highlight the limitations and the need for alternatives to currently available assays (*30*–*32*). Despite sharing a common N terminal region with GP1,2 and the demonstrated cross-reactivity of antibodies, sGP's potential for identifying vaccine-induced immunity remains largely unexplored. In this study, we tested samples from 48 ERVEBO vaccinees from the United States. All vaccinee samples tested positive with EBOV sGP (100% sensitivity, 97.6% specificity), demonstrating that sGP effectively detects antibody responses to EBOV GP1,2. In contrast, EBOV GP1,2 testing detected antibodies in 46 vaccinees (95.8% sensitivity, 98.8% specificity), indicating that sGP not only detects antibodies to EBOV GP1,2 but outperformed GP1,2.

Reliable identification of vaccine-induced immunity is essential for monitoring immune durability, assessing vaccine efficacy, and detecting breakthrough infections. To differentiate vaccine-induced immunity, one strategy is to target viral antigens absent from current vaccine formulations; another is to detect immune responses against the vaccine vector itself. A previous study addressing the strategy to target viral antigens developed an assay by using peptides from viral protein (VP) 40, VP35, and NP, viral antigens not included in the current EBOV vaccines (*35*). Two studies investigating vector immunity reported suboptimal responses, with only 28% seroconversion to VSV-M at 56 days postvaccination, no response to VSV-M, and only 1 vaccinee with VSV-N antibodies in another study (*36*,*37*). In this study, we show that VSV-P-N antibodies are detected in all vaccinees. Of note, similar to sGP, VSV-P-N detected vaccinees that were missed by GP1,2, highlighting its added sensitivity. Although the samples were limited to US vaccinees, we did not observe a decline in either assay sensitivity or median signal intensity. That observation contrasts with a previous study that reported waning VSV-N responses over time, with a corresponding decrease in assay sensitivity from 90% to 65% 1 year after vaccination (*38*). Further studies with larger longitudinal cohorts are needed to confirm those findings. Overall, our results support the utility of this assay for long-term monitoring of immune responses to both the target antigen and the vaccine vector.

A study conducted during the 10th EVD epidemic in DRC (*8*) described the protective efficacy of ERVEBO vaccination against death in patients with confirmed EVD. The study reported a 25% case-fatality rate among vaccinated patients, contrasting with the 100% vaccine efficacy previously reported (*7*). Although an inadequate immune response because of host-related factors, logistical issues, and administration errors could explain the discrepancy between the 2 studies, potential misclassification of vaccination status might have contributed to the differences observed. Without reliable assays, such insights depend solely on self-reported vaccination status. Our results from the Guinea cohort with samples from persons who had a vaccination card revealed that VSV-P-N and EBOV sGP double positivity confirmed the ERVEBO vaccination status of 94.8% of persons. In contrast, the percentage of double positivity by using EBOV GP1,2 was much lower at 71.3%. Similarly, a greater number of persons who had verbally confirmed their vaccination status were identified by double positivity for VSV-P-N and EBOV sGP (VSV-P-N and EBOV sGP 90.8%, VSV-P-N and EBOV GP1,2 63.3%). Those results underscore the value of this assay in verifying vaccination status and support the use of VSV-P-N with EBOV sGP as a superior alternative to EBOV GP1,2 for detecting vaccine-induced antibody responses. 

A 2023 study assessed the seroreactivity of 698 samples from healthcare providers and frontline workers to EBOV GP1,2 by using both FANG ELISA and a Luminex assay targeting additional antigens (*39*). The study reported that 1.4% of samples were reactive to >1 EBOV antigen with the Luminex (Diasorin) assay. Of note, only 0.8% of the samples were positive by both FANG ELISA and Luminex GP1,2, despite individual positivity rates of 7% for FANG ELISA and 9.4% for Luminex GP1,2, highlighting poor agreement between the 2 assays. The study was conducted at Boende General Hospital in Tshuapa Province, whereas samples used in our study were collected from healthcare workers in Tshuapa territories outside the Boende health zone. Consistent with the 2023 study, we found only 1 sample reactive to both EBOV sGP and GP1,2, corresponding to a seroreactivity rate of 1.3%.

The first limitation of our study is the absence of samples from unvaccinated EVD survivors, which would have provided further insights into the assay’s ability to differentiate between vaccine-induced and infection-acquired immunity. Because survivors typically exhibit higher GP antibody levels than vaccinated persons, we anticipate that the assay would demonstrate high sensitivity for detecting survivor antibodies. However, comprehensive differentiation between vaccination and natural infection would require incorporation of additional viral antigens such as NP and VP40, because antibody responses to those antigens are typically observed only after natural infection. An additional limitation of this study is that the assay is specific to identifying ERVEBO vaccination status. Future studies incorporating survivor samples and expanded antigen panels will be essential for complete assay validation and epidemiologic applications.

In conclusion, our study introduces a novel multiplex serologic assay that includes EBOV sGP and VSV-P-N antigens to improve detection of postvaccination antibodies. The assay demonstrates high sensitivity and specificity and requires minimal sample volume. With the assay’s increased sensitivity, this platform could strengthen the evaluation of vaccine-induced immunity by helping identify correlates of protection, assess the durability of immune responses, and support adverse event reporting by differentiating vaccine-related reactions from symptoms of concurrent infection. By using samples from US ERVEBO vaccinees and post–ring vaccination samples from Guinea, we found that sGP offers superior sensitivity compared with GP1,2, and that the combined use of sGP and VSV-P-N reliably identifies vaccinated persons.

Appendix Additional information about confirming ERVEBO vaccination to support Ebola virus surveillance.
