# Supplementary material for: Confirming ERVEBO Vaccination to Support Ebola Virus Surveillance
Source: Emerg Infect Dis. 2026 Apr;32(4):533–42. doi: 10.3201/eid3204.251906 (PMC13094833; doi:10.3201/eid3204.251906)
Supplement: Appendix — Additional information about confirming ERVEBO vaccination to support Ebola virus surveillance. [file 25-1906-Techapp-s1.pdf]

Article DOI: https://doi.org/10.3201/eid3204.251906

EID cannot ensure accessibility for supplementary materials supplied by authors. Readers who have difficulty accessing supplementary content should contact the authors for assistance.

Confirming ERVEBO Vaccination to Support Ebola Virus Surveillance

Appendix

Supplementary Material

Development of Multiplex Luminex Assay

To detect antigen-specific antibodies, a bead suspension containing 500 beads of each protein type in 50 µl/well was prepared and transferred to the wells of a 96-well black plate (ThermoFisher). Test samples were prepared at a 1:50 dilution in 50 µl of Assay Buffer and transferred to the plate. The sample-bead mixture was incubated for 2 hours at room temperature on a plate shaker at 800 rpm and washed three times with 150 µl/well Wash Buffer, followed by a final wash with 150 µl/well Assay Buffer. Anti-human IgG-PE (9040-09, SouthernBiotech) detection antibody was prepared in Assay Buffer at a 1:1000 dilution and 50 µl of antibody added to the wells. Following 1 hour of incubation at room temperature on a plate shaker, wells were washed as described above with a final wash in Luminex Sheath Fluid (ThermoFisher) and resuspended in 60 µl/well Luminex Sheath Fluid. The plates were analyzed on Luminex xMAP Intelliflex (ThermoFisher). The results were analyzed as median fluorescence intensity (MFI) of 50 beads for each bead region per well.

Internal Reference Standard

A serum sample from a vaccinee who received the rVSV-EBOV vaccine was mixed 1:1 with EBOV-infected nonhuman primate (NHP) serum to generate an internal reference standard. A two-fold dilutions of internal reference standard, ERVEBO vaccinee serum, and EBOV-infected NHP serum ranging from 1:100-1:102,400, were run in parallel with the same dilution ranges of Filovirus Animal Nonclinical Group (FANG) ELISA third-generation reference sample (BMIZAIRE108), low quality control sample (Low QC-BMIZAIRE109), high quality control sample (High QC-BMIZAIRE110), and negative control (BMI529) (kindly provided by Lisa

Hensley), and ELISA Units/ml of the internal standard was interpolated using a 4-parameter logistic (4PL) curve (27). The internal control is included in all assay plates as a 10-point, 2-fold serial dilution series and used for normalization to correct for inter-plate variation (Figure S2). All MFI readings are background-corrected by subtracting the mean MFI of the duplicate normal human sera samples on each plate.

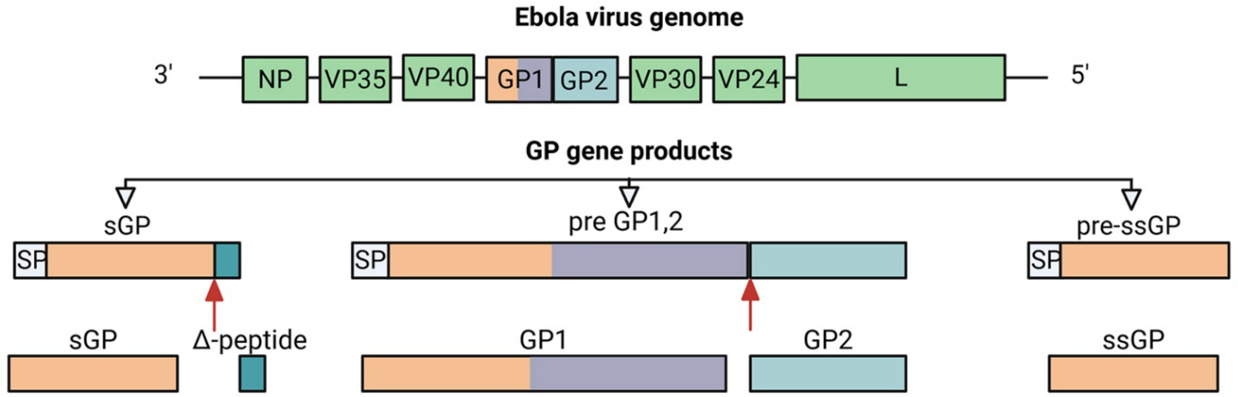

Appendix Figure 1. The products of glycoprotein gene of ebolaviruses. Schematic illustration of the GP gene products of the EBOV genome. Site-specific transcriptional editing of the glycoprotein gene gives rise to three products with identical N-terminal 295 amino acids (indicated in orange): GP1,2, soluble GP (sGP), and small soluble GP (ssGP). The red arrow markes the cleavage site: SP: signal peptide.

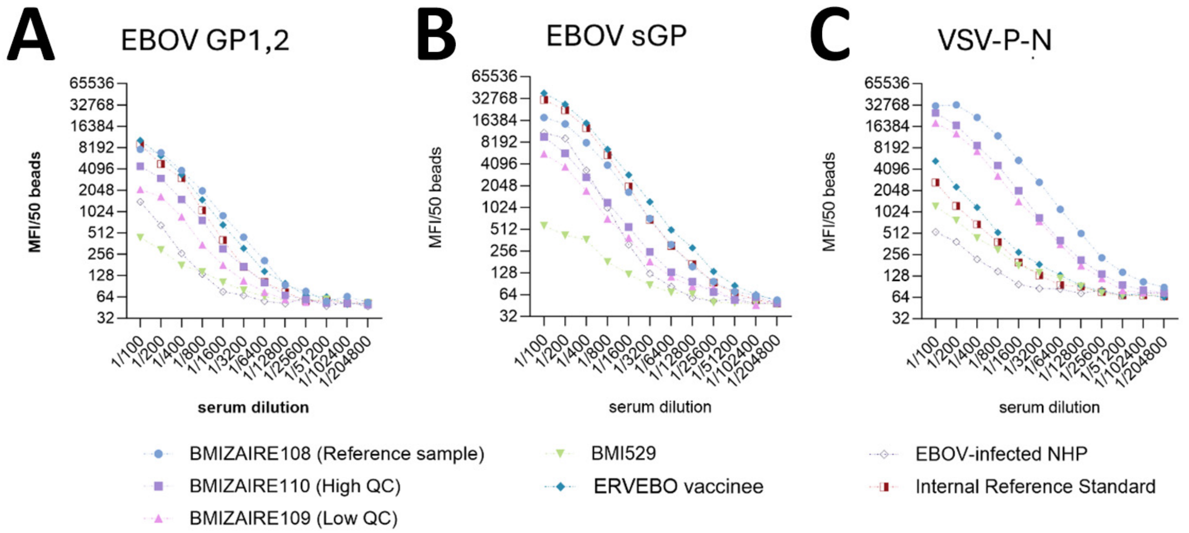

Appendix Figure 2. Assessment of internal reference standard and FANG ELISA standards. FANG ELISA reference samples BMIZAIRE 108, BMIZAIRE 109, BMIZAIRE 110, BMI529, and serum samples

from ERVEBO vaccinee, EBOV-infected NHP, and internal reference sample (1:1 mix of serum samples from ERVEBO vaccinee, EBOV-infected NHP) were tested in 2-fold dilution series starting at 1:100 with the multiplex Luminex assay. MFI reads from A) EBOV GP1,2, B) EBOV sGP, and C) VSV-P-N are presented.

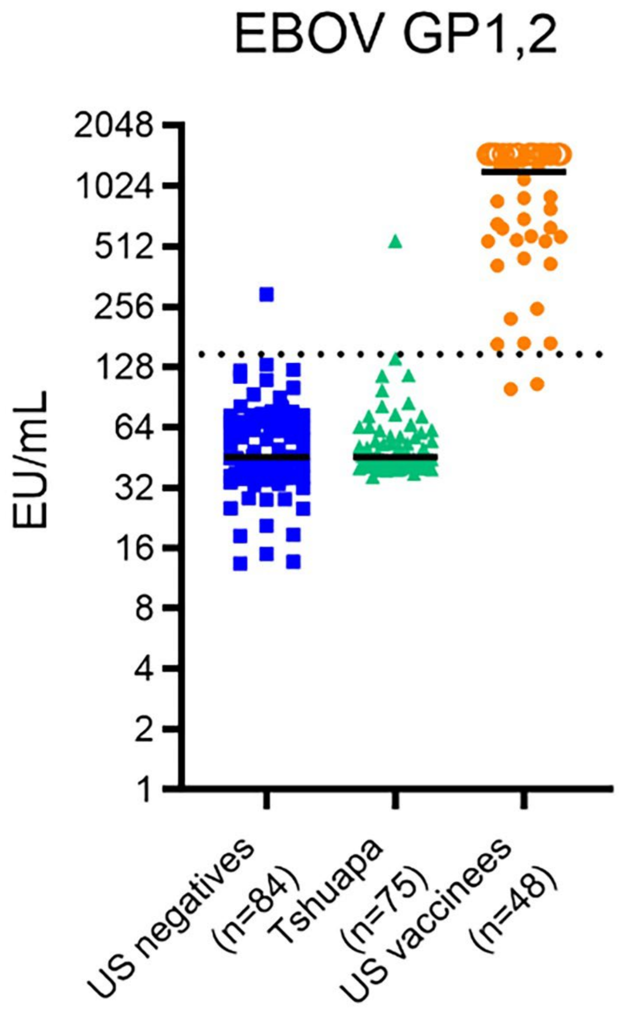

Appendix Figure 3. Detection of EBOV GP1,2 antibodies in ERVEBO vaccinees. EBOV GP1,2 was used to detect antibodies, and results were represented as ELISA Units/mL (EU/mL) using 4PL curve generated with the internal reference standard. The EU/mL of the internal reference standard was determined to be 1024 EU/mL using FANG ELISA reference samples. Open orange circles in US vaccinee samples represent samples that reached the upper detection limit.

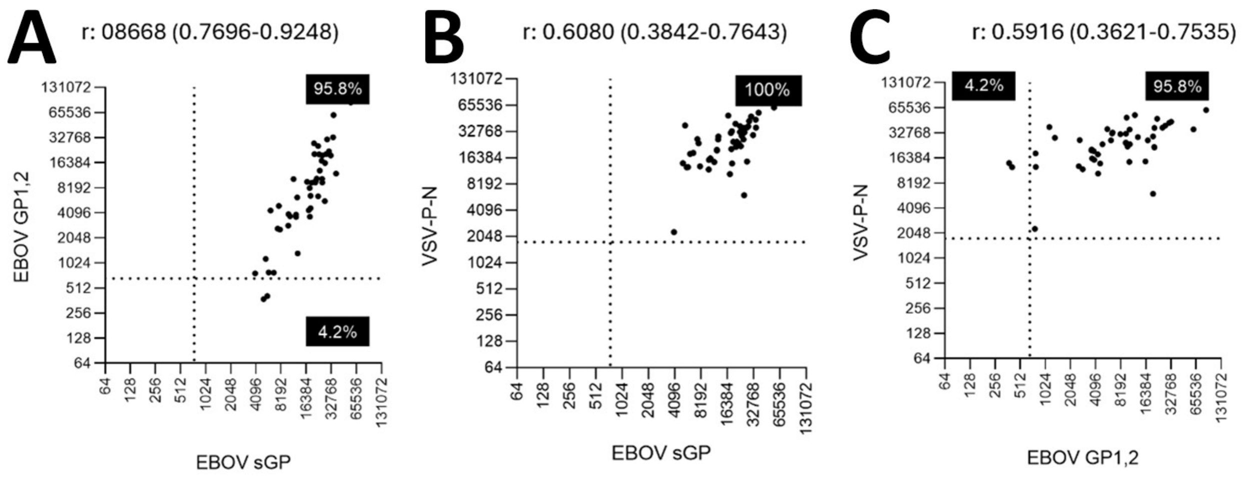

Appendix Figure 4. Correlation analysis of US vaccinee samples tested with EBOV GP1,2, EBOV sGP, and VSV-P-N. Spearman correlation analyses were performed between A) EBOV GP1,2 and EBOV sGP, B) VSV-P-N and EBOV sGP, and C) VSV-P-N and EBOV GP1,2 with US ERVEBO vaccinees.

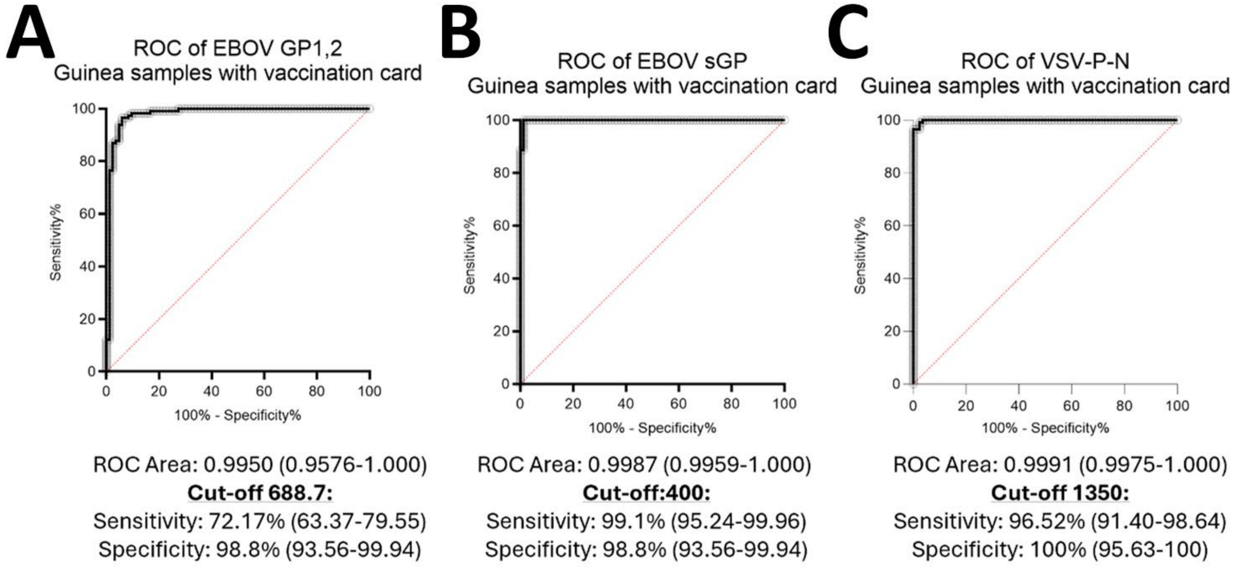

Appendix Figure 5. ROC analysis of EBOV GP1,2, EBOV sGP, and VSV-P-N using samples from individuals with vaccination cards. ROC analysis performed for A) EBOV GP1,2, B) EBOV sGP, and C), VSV-P-N using samples from 115 individuals who presented a vaccination card as true positives. ROC area, assay sensitivity, and specificity were given with 95% confidence intervals.
